# The Queen's Jubilee Hospital

**Published:** 1895-08-10

**Authors:** 


### THE INSTITUTIONAL WORKSHOP.

### HOSPITAL ADMINISTRATION.

### THE QUEEN'S JUBILEE HOSPITAL.

Hearing that the Distribution Committee of the Hospital Sunday Fund, after an interview with the committee of the Jubilee Hospital, at which the auditors, Messrs. Whinney and Co., were present, had determined not to recommend a grant to this institution, we decided to pay it a visit and judge for ourselves. Accordingly, on Saturday, July 20th, we called at the hospital in the old Brompton Road about six p.m., and asked for the matron. We were courteously received by her, and taken over the building, which is in reality an old dwelling-house with a fair amount of ground behind it. The building appears to consist of two storeys and a basement. The basement contains the kitchens, dispensary, and offices, and the ground floor has the board-room, secretary's office, and out-patient department, the latter consisting of two rooms, the doctors sitting in one, and the out-patients in the other. On the first floor there are three small bed-rooms, one of which is used as the matron's bed and sitting room, the second as a sleeping-room for two nurses, and the third as a one-bedded ward. There are, in addition, two larger rooms, which are literally filled with beds. That is to say, there are six in each room, although at the outside not more than three—and probably not more than two—beds ought to be placed in either of them. There is one w.c. and one bath-room for male and female patients, and one slop-sink which, we gathered, was common to both male and female wards. The building is in very bad decorative repair inside and out; and, although we made no inspection of the drains, the atmosphere of the place left us with a strong impression that it is decidedly insanitary. We have applied to the hospital authorities for permission to make a plan of the institution, showing exactly how the beds are placed, and, when reduced in number, how they might be placed with some regard to the wellbeing and comfort of the inmates. It is altogether improper to call this old house under present circumstances a hospital, and to endeavour to make the public believe that it is one of the general hospitals of the metropolis.

### AN UNEXPECTED INTERVIEW.

After talking to the matron, from whom we learned that the nursing staff consisted of herself, one trained nurse, and two probationers, one of the latter having to do all the night duty for thirteen patients, some of them serious accident cases, we were asked if we would see the secretary. Desiring to afford him every opportunity of explanation or suggestion, we consented to go and see him, and the matron invited us to go downstairs to his office. We were shown into the board-room, where we found a committee proceeding, at which there were present, besides two members and the secretary, a deputation of four working men, representing some large works in the district. The conference was discussing the best means of organising a demonstration and fête in aid of the hospital. It was certainly a surprise to be thus suddenly shown into the committee-room, and we determined to utilise the occasion to arrive at a just judgment as to the true inwardness of the institution and its management. The interview, which lasted for quite an hour, briefly amounted to this. The Chairman, Mr. Benham, maintained that although the two large wards contained twice the number of beds which they ought to, the results were excellent. That patients preferred to go into one of the beds of the Jubilee Hospital to going into any hospital bed anywhere in London. He said that all they wanted was more money, and that if the Hospital Sunday Fund or the public would supply the committee with larger funds they would at once set about building an out-patient department at the back of the hospital, and then some of the beds could be moved out of the crowded wards into the rooms on the ground floor now occupied by this branch of the work. He informed us that there was no house surgeon, but that one of the medical staff was accessible in cases of emergency, and could be fetched to the hospital within ten minutes of the arrival of a severe case. On arrival during the daytime the patient was received, and in case of urgent necessity, such as hæmorrhage, was attended to by the "Surgical" porter, who had had several years' experience at the Orthopædic Hospital, and had been at the Jubilee Hospital from the date of its opening. That during the night time, pending the arrival of the doctor, everything that was necessary was done for the patients by the secretary, who resided on the premises. He further said that there was no law against treating or operating upon patients in a cellar, or under any conditions, however unfavourable, if a doctor chose to do so, providing the patient recovered, though if he died, he admitted that the coroner's inquest might bring about unpleasant results. Mr. Benham further stated that Sir Sydney Waterlow, at the interview the committee had with him, had said that the cost per bed at the hospital was excessive at the present time, although they had thirteen beds, and all available space was occupied by them. The secretary stated that the cost of a bed was about £78 per annum, or thirty shillings a week. He further urged that it would never do to reduce the number of beds, as such a step would nearly double the cost per bed at which the institution was maintained, and so cause much stronger objection to be made against the management of the hospital. He further stated that they attended several hundreds of out-patients in the course of the year, and that he made inquiry into their condition and found that seven out of nine of them were painters out of work with their wives and children, who were absolutely unable to pay anything for their medical treatment. Asked whether the hospital committee would permit a proper plan to be made of the whole institution as it stood that day, free of cost to the hospital, providing a duplicate of the plan was presented to the institution, Mr. Benham expressed a hope that the suggestion was not made with a view to the publication of the plan. The working men present testified to the kindness which had been extended to their fellow workmen who had met with accidents, and had attended the institution as out-patients. They declared that the West London Hospital was very unpopular with the working classes of the district, and that it was essential to have further hospital accommodation in the neighbourhood without delay. Other points were raised which have no special bearing upon this institution, and we therefore omit them.

An Expert's Opinion.

Our reply, which was apparently assented to by all present except Mr. Benham, who did not commit himself, was briefly as follows. If Miss Nightingale could visit the Jubilee Hospital and its wards as they appeared that day, she would probably declare that their condition was little, if any, better than those of the Scutari Hospital in its worst days during the Crimean War. It was distinctly wrong to place six beds in any of the wards, as three was the very outside number which they could accomodate. To do so cast a heavy responsibility upon the medical staff, and rendered it impossible for any honest man, with a knowledge of hospital requirements and hygiene, to support or countenance the continuance of the Jubilee Hospital. Certainly no independent body of gentlemen like the council of the Hospital Sunday or Hospital Saturday Funds, could make a grant to the institution in the state in which it was that day, for the sufficient reason that if they did they would become *participes criminis*. If Mr. Benham and his committee would permit us to make an accurate plan of the hospital and its wards, showing the position of the beds and the superficial area and cubic space, and if that plan were placed upon the committee-room table it would show the least experienced committee man how improper it was to receive patients under present conditions. Mr. Benham's cellar argument was remarkable and instructive. Persons could commit offences punishable by law, or they might commit a more serious offence though it did not come within the law, as Mr. Benham pointed out, namely, the iniquity of undertaking a public duty, the adequate provision for the care of the sick, and then neglecting it by placing patients, as in that institution, under conditions which tended to delay, if not to defeat, recovery.

The committee must take the responsibility of deciding whether they would continue the present evils so patent to the eyes and senses of the visitor, or whether they would determine to regard the work they asked the public to give them funds to undertake (for we desire to cast no reflection upon the good intentions of anybody connected with the institution) with the single intention of providing that it should be done as perfectly and thoroughly as possible. If they took the latter course and showed that they had effected a revolution in the present management, by making the institution a cottage hospital with five beds, which was the best that could be done with it as it stood, in due course they would reap their reward. If at any rate they determined to perpetuate the overcrowded insanitary wards (and if the working men present wanted to know what those wards meant to the patients they should visit them about two a.m.), then it was certain that the institution must be closed, as its continuance was not calculated to promote the public welfare or to minister adequate treatment to the sick who might be induced to enter it. We asked them to let us know whether or not they were prepared to permit a plan to be made and the whole truth revealed to the committee and all interested in the hospital.

We had purposely held aloof from the controversies which had arisen round the Jubilee Hospital, had visited it independently in order to see how and what work was being done. We welcomed the opportunity of pointing out the present condition of affairs to the committee themselves before making the result of our inspection public as it must be made public, so as to secure that the patients on the one hand were protected from unnecessary risks, and that the public on the other might have the opportunity of learning the present state of affairs, and so judging for themselves whether they are justified in giving money to enable the evils pointed out to be perpetuated at the expense of the ignorant and innocent sick poor of the neighbourhood. In our judgment it is a calamity that a gentleman who deservedly won the respect of so many members of the profession during his curacy at All Souls', Langham Place, as the Rev. W. R. Mowl did, should continue to allow his name to be advertised as the chairman of the committee of the Jubilee Hospital, having regard to the truly startling conditions in which it is at present. We urge the committee to have regard to the Christian duty of seeing that no sick person is admitted to treatment, except he be secured within the building hygienic conditions, and the opportunity for making a speedy recovery far superior to any which the Jubilee Hospital now provides.

